# Statistical shape modeling of the proximal femur in Mexican women: a cross-sectional morphometric and densitometric study

**DOI:** 10.1007/s11657-026-01682-0

**Published:** 2026-04-24

**Authors:** Griselda-Adriana Cruz-Priego, Fryda Medina-Rodríguez, Rubén Torres-González, Patricia Clark

**Affiliations:** 1 Faculty of Medicine of National Autonomous University of Mexico (Universidad Nacional Autónoma de México), Mexico City, Mexico; 2 Clinical Epidemiology Research Unit, Hospital Infantil de Mexico “Federico Gomez”, Mexico City, Mexico; 3 Cochrane UNAM-Mexico Group, Mexico City, Mexico; 4 Unidad Médica de Alta Especialidad (UMAE), “Dr. Victorio de la Fuente Narváez”, Instituto Mexicano del Seguro Social (IMSS), Ciudad de Mexico, Mexico; 5 Dirección de Educación e Investigación en Salud de la UMAE de Traumatología, Ortopedia y Rehabilitación “Dr. Victorio de la Fuente Narváez”, IMSS, Mexico City, Mexico

**Keywords:** Femoral morphology, Osteoporosis, Statistical shape modeling, Bone mineral density, Hip fracture, Mexican population

## Abstract

***Summary*:**

This study applied statistical shape modeling to 284 DXA hip scans from Mexican women, identifying proximal femur morphotypes characterized by long, narrow necks and valgus orientation. These shape patterns showed directionally lower regional BMD in vulnerable areas, although associations were modest. The findings describe femoral configurations that resemble those previously associated with fracture risk in international cohorts, supporting the potential role of morphometric analysis as a complementary, population-specific approach to skeletal fragility assessment.

**Purpose:**

Hip fractures are a major cause of disability and mortality in older adults, particularly among women with osteoporosis. Bone mineral density (BMD) is a cornerstone of fracture risk assessment but does not fully capture biomechanical and structural vulnerability. This study is aimed at characterizing proximal femur morphology in Mexican women using statistical shape modeling and at examining its relationship with regional BMD.

**Methods:**

In this exploratory cross-sectional analysis, 284 dual-energy X-ray absorptiometry (DXA) hip scans were processed with BoneFinder software to generate morphometric profiles. Active Shape Models (ASM) identified the main modes of shape variation, which were analyzed in relation to site-specific BMD.

**Results:**

Seventeen femoral configurations were identified, with three representative morphotypes: long, narrow necks with valgus alignment, short and wide necks, and intermediate morphologies. Shape patterns characterized by long, narrow, valgus-oriented femoral necks showed directionally lower regional BMD in the superior neck and Ward’s triangle—sites critical for fracture initiation—although associations were modest in regression analyses. Automated morphometry identified structural traits linked to mechanical disadvantage beyond traditional cortical indices.

**Conclusion:**

This is the first study to apply statistical shape modeling to Latin American women, identifying recurring femoral shape patterns that resemble morphologies previously associated with fracture risk in international cohorts. While prospective validation with fracture outcomes is needed, this exploratory study provides foundational evidence for anatomically informed, population-specific approaches to skeletal fragility assessment.

**Supplementary Information:**

The online version contains supplementary material available at 10.1007/s11657-026-01682-0.

## Introduction

Hip fractures are among the leading causes of morbidity and mortality in older adults, largely due to the increased fragility of the proximal femur caused by osteoporosis [1–5]. Although tools like FRAX [6] have been developed to estimate fracture risk, their predictive accuracy has been questioned, as they often exclude biomechanical variables critical to understanding fracture mechanisms. Bone mineral density (BMD) remains a cornerstone of fracture risk assessment, but it is insufficient on its own to predict failure load or structural integrity.

Fractures result not only from low BMD but also from how mechanical forces are transmitted through the bone [7, 8]. Biomechanical variables—especially those related to bone geometry—play a fundamental role in skeletal strength [7–12].

Parameters such as femoral neck width, hip axis length, and the cervico-diaphyseal angle have been associated with fracture risk, yet these isolated measurements often exhibit collinearity and limited independent predictive power [8, 13–15]. To address this limitation, morphometry has emerged as a robust technique for analyzing complex anatomical variations of the femur [16–18].

Morphometry provides a multidimensional profile of femoral morphology that captures subtle shape differences, which may correlate with biomechanical vulnerability [7]. Studies combining shape analysis with BMD data have demonstrated superior predictive performance compared to traditional models based solely on densitometry. Notably, studies [17, 19–22] showed that femur shape-enhanced models significantly improve fracture risk discrimination among postmenopausal women.

Previous studies in Mexican populations, such as those by Balderrama et al. [23] and Gómez García (1995) [24], explored radiographic indices like the cortical index of Lizaur (ICD) and the femoral cortical index of Gómez (ICDF) to estimate bone quality. While based on simpler geometric measurements, these works provide a valuable comparative foundation for current morphometric approaches.

Despite these advancements, most morphometric studies have focused on European or North American populations, leaving important gaps in our understanding of femoral morphology in other ethnic groups. Population-specific data are essential for refining predictive algorithms and ensuring their applicability across diverse settings.

To our knowledge, no study has yet applied statistical shape modeling to DXA scans in Latin American populations. This exploratory work aims to fill that gap by providing baseline data for future prospective studies. Specifically, we sought to characterize the morphological variation of the proximal femur in a sample of Mexican women using ASM and to examine how shape variations relate to biomechanical vulnerability and fracture risk. By integrating morphometric and densitometric data—particularly from Ward’s triangle—this study contributes essential evidence for the development of culturally adapted and clinically relevant risk prediction tools.

## Methods

This was a cross-sectional descriptive pilot study conducted using dual-energy X-ray absorptiometry (DXA) hip scans from adult patients. Images were sourced from the DXA image database at the Clinical Epidemiology Research Unit of the Hospital Infantil de México (through 2017 and 2018). The objective was to characterize bone architecture in the proximal femur through morphometry.

Adult patients were included if they had proximal femur DXA images accompanied by BMD reports. DXA images were obtained with a LI43616ES iDXA bone radiodensitometer (Lunar-GE). Exclusion criteria included images that lacked sufficient quality for analysis (e.g., poor contrast or incomplete visualization of bone contours).

Based on the estimation approach for morphometric shape modeling, a minimum of 200 images was deemed necessary to establish a robust statistical shape model, in line with the methodology proposed by Cárdenas and Correa [25].

Hip shape analysis was performed using BoneFinder software, a fully automatic system developed at the University of Manchester [26, 27]. The software detects the proximal femur in DXA images using the Random Forest Regression Voting (RFRV) framework [28], places 65 anatomical landmarks, and internally constructs the Statistical Shape Model (SSM). Landmark placement was performed automatically by BoneFinder using the Random Forest Regression Voting (RFRV) framework described by Lindner et al. [28, 29] and Cootes et al. [30]. This method has been extensively validated for accurate capture of proximal femur contours on DXA and radiographic images. As part of our quality control procedure, all segmentations were visually inspected to ensure appropriate anatomical landmark placement.

Following the standard Active Shape Model methodology described by Cootes and Taylor [29, 30], the SSM is generated by training PCA on a set of aligned shapes, where alignment removes differences in position, orientation, and scale prior to extracting morphological variation. This procedure yields the mean femur configuration (mode 0) and a set of orthogonal modes of variation (hip shape models, HSMs) that describe independent patterns of proximal femur morphology. Subject-specific mode values for all HSMs were obtained directly from the BoneFinder automatic output.

The study was conducted using retrospective, anonymized data and followed institutional ethical guidelines for secondary analysis of clinical images.

### Statistical analysis

Descriptive statistics were performed after assessing data distribution. Continuous variables are presented as means with standard deviations, as appropriate. Hip shape modes (HSMs) were treated as continuous standardized principal component scores.

Exploratory linear regression analyses were conducted to evaluate associations between individual HSM scores and regional BMD measurements at the femoral neck, upper neck, lower neck, Ward’s triangle, and trochanteric regions. Given the cross-sectional and exploratory nature of the study, no multivariable adjustment for potential confounders was performed.

Representative morphometric patterns were derived using the statistical shape modeling framework implemented in BoneFinder and are presented for descriptive and illustrative purposes only; these patterns do not correspond to groups of individual participants.

All statistical analyses were performed using R version 4.5.0 (R Core Team, 2025). No missing data were present in the analyzed sample.

## Results

A total of 284 women were included, with a mean age of 54 years. Morphometric analysis using BoneFinder software identified 17 distinct morphological configurations of the proximal femur, consisting of a baseline configuration (mode 0) and 16 additional modes.

Descriptive characteristics of the study population are presented in Table 1. The sample had a mean height of 153.9 cm, mean weight of 63.4 kg, and mean BMI of 28.8 kg/m^2^. Mean values for hip shape modes (HSM0–HSM17) were close to zero, as expected for standardized principal component scores.

**Table 1:** Descriptive characteristics of the study population (*n* = 284)

Variable	Mean (SD)
Age (years)	54.0 (9.10)
Height (cm)	153.86 (5.99)
Weight (kg)	63.36 (0.95)
BMI (kg/m^2^)	28.8 (6.22)
HSM0	0.0001 (0.0385)
HSM1	0.0000 (0.0335)
HSM2	0.0000 (0.0339)
HSM3	0.0001 (0.0348)
HSM4	0.0000 (0.0334)
HSM5	0.0000 (0.0332)
HSM6	0.0000 (0.0331)
HSM7	0.0000 (0.0330)
HSM8	0.0000 (0.0332)
HSM9	0.0000 (0.0330)
HSM10	0.0000 (0.0330)
HSM11	0.0000 (0.0330)
HSM12	0.0000 (0.0331)
HSM13	0.0000 (0.0331)
HSM14	0.0000 (0.0331)
HSM15	0.0000 (0.0331)
HSM16	0.0000 (0.0330)

HSM0–HSM16 are standardized principal component scores from the statistical shape model; their means are approximately zero by construction

**Fig. 1:**
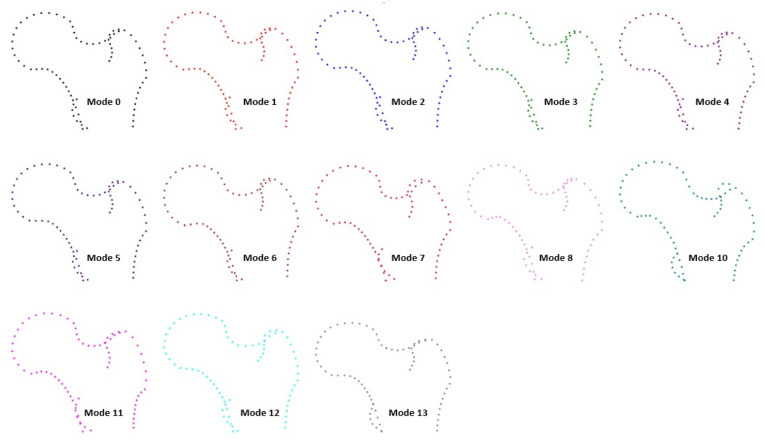
Proximal femur morphometry. Each mode is illustrated as a representative shape configuration derived from variation along the corresponding principal component. The first 5 modes (mode 0–mode 4) capture 80% of the variability in bone morphology observed in the sample

The first five modes (modes 0–4) accounted for 80% of the total shape variability, and the first 13 modes captured 95% of the variability observed in the sample (Fig. 1). Accordingly, Fig. 1 displays only the first 13 modes, which capture the vast majority of shape variation, while higher-order modes explaining minimal variance were not illustrated.

Three primary femoral neck morphologies were descriptively identified: (1) long and narrow necks in modes 2, 6, and 12; (2) short and wide necks in modes 3, 7, and 10; and intermediate shapes in modes 0 and 13. Long, slender necks showed greater head–diaphysis separation, whereas short, broad necks demonstrated reduced separation.

The femoral head also exhibited morphological variation. Modes 1, 2, 4, and 10 featured a more prominent and spherical head, whereas modes 6, 8, and 13 showed a flatter and less curved head shape. The cervico-diaphyseal angle also varied across configurations: modes 2, 10, and 12 demonstrated a more open (valgus) angle, while modes 7 and 8 exhibited a more closed (varus) alignment. Greater trochanter expansion was more pronounced in modes 3, 5, and 7, whereas modes 10 and 11 showed a more inferior and medial positioning of the lesser trochanter.

Quantitative measurements supported these morphological distinctions. Femoral neck length was greatest in modes 2, 6, and 12, whereas neck width was broader in modes 7 and 10 and narrower in mode 2. The cervico-diaphyseal angle was largest in valgus-type morphologies and smallest in varus types. Femoral head diameter and intertrochanteric width also varied across modes, consistent with the observed patterns of proximal femoral morphology.

Illustrative BMD values for the representative morphological patterns generated by the statistical shape model (mode 0–mode 5) are provided in Supplementary Table 1. These configurations represent mean shape patterns and do not correspond to patient groups.

BMD descriptive analysis showed that the lowest values were consistently observed in the superior neck and Ward’s triangle regions. While illustrative values from the representative shape configurations suggest that the prototype corresponding to mode 2 is associated with lower BMD in these regions, this pattern was not supported by the regression estimates, as confidence intervals included the null value and the corresponding *p*-values were large.

Exploratory linear regressions were performed to evaluate the relationship between each hip shape mode and regional BMD. Most associations were small in magnitude, with confidence intervals frequently spanning the null value. Modes characterized by long and narrow femoral necks tended to show lower BMD in the upper neck and Ward’s triangle, whereas modes with shorter and wider necks showed small positive associations in some regions. Because the full set of regression outputs is extensive, all coefficients, standard errors, confidence intervals, and p-values for the 17 modes across all BMD regions are provided in Supplementary Table 2. Overall, the analyses show that morphological variation in the proximal femur is accompanied by modest regional differences in BMD.

## Discussion

Early studies in Mexican cohorts, such as Clark et al. [31], investigated clinical and radiographic correlates of hip fracture but did not characterize geometric variation in the proximal femur. To our knowledge, this is the first study to apply statistical shape modeling (SSM) to DXA images in Latin American women, providing exploratory morphometric data from an underrepresented population and offering a novel perspective on skeletal fragility in this demographic.

The present findings highlight the relevance of proximal femur geometry for structural integrity. Mode 2, characterized by a long and narrow femoral neck, a flatter head, and a valgus cervico-diaphyseal angle, closely resembles morphotypes previously associated with increased fracture susceptibility in European and North American cohorts. Notably, this valgus-oriented configuration appeared relatively frequent in our sample, suggesting potential population differences that warrant further examination. Mode 3, in contrast, exhibited a shorter and wider neck with lateral trochanteric expansion—features that may improve load distribution but that have also been linked to increased cortical stress in the intertrochanteric region. Mode 0, with intermediate geometry, offers a biomechanically neutral reference for interpreting these deviations.

Exploratory regressions demonstrated modest but directionally consistent associations between femoral shape and regional BMD. Long, narrow, valgus-oriented morphologies tended to show directionally lower BMD estimates at the upper neck and Ward’s triangle, whereas shorter and wider shapes showed small positive trends. These patterns support the biological plausibility of the morphotypes identified and justify further evaluation in longitudinal or case-control studies [19].

Comparisons with international literature emphasize the novelty of these findings. European SSM studies have shown that femoral shape enhances fracture discrimination beyond BMD alone [16, 19, 21, 22]. However, the geometric features most strongly linked to fragility—long slender necks, flatter heads, valgus alignment—may vary in prevalence across populations. Epidemiological data indicate that Mexico has intermediate hip fracture rates relative to Europe and Asia, raising the possibility that population-specific femoral morphology may contribute, alongside metabolic and environmental factors, to observed differences in fracture incidence. The present results provide an initial morphological framework for understanding these patterns in Mexican women.

Recently, Scott et al. reported a large longitudinal analysis of DXA-derived hip shape and incident hip fracture in the UK Biobank [32], demonstrating that specific shape modes—particularly those characterized by a narrower femoral neck and increased neck–shaft angle—were independently associated with hip fracture risk. While that study focused on fracture prediction in a predominantly European population, our findings complement this work by providing population-specific morphometric data in Mexican women and by exploring the relationship between femoral shape and regional BMD in a cross-sectional setting.

This study offers several methodological strengths, including the integration of SSM with regional BMD, the use of automated landmark detection via BoneFinder, and a sample size adequate for modeling 17 shape modes. Nonetheless, some limitations must be acknowledged. The cross-sectional design precludes causal inference, and the absence of fracture outcomes limits evaluation of predictive performance. In addition, the DXA images were obtained from a single center, generalizability may be restricted. Additionally, DXA provides lower spatial resolution than radiographs for assessing cortical features such as thinning or periosteal expansion, though this limitation also points toward opportunities for radiograph-based morphometric studies in broader clinical settings.

Although this study cannot identify individuals at increased fracture risk, the characterization of morphotypes with less favorable geometric and densitometric features may inform future work on early preventive strategies. Independent of morphology, substantial evidence shows that resistance training, multimodal exercise, and balance-focused programs improve lower-limb strength and reduce fall incidence and fracture risk in older adults [33–37]. These established benefits underscore the potential role of anatomical profiling in complementing preventive efforts, particularly for individuals who may present structural configurations associated with mechanical vulnerability in prior research.

Overall, integrating femoral shape analysis with regional BMD appears to be a promising approach for improving the anatomical characterization of skeletal fragility. While prospective validation is required before clinical application, these exploratory findings underscore the importance of incorporating geometric phenotype—alongside traditional densitometric measures—when developing fracture risk models tailored to Latin American populations.

## Conclusion

This study documents, for the first time in a Latin American population, the existence of distinct proximal femur morphological patterns associated with relevant biomechanical and densitometric variations. In particular, configurations with long, thin, valgus-oriented necks and flattened femoral heads were identified as having greater theoretical susceptibility to subcapital fractures.

Ward’s triangle was also confirmed as a region of low bone mineral density and high vulnerability, reinforcing its clinical value in assessing fracture risk. Integrating morphometric and densitometric data improves the understanding of bone fragility and lays the foundation for developing more accurate and culturally relevant predictive models for Latin American populations.

## Supplementary Information

Below is the link to the electronic supplementary material.

ESM 1 Supplementary Material 1 (DOCX 16.0 KB)

ESM 2 Supplementary Material 2 (DOCX 25.2 KB)

## Data Availability

All data generated or analyzed during this study are fully reported within the manuscript.
