# Supplementary material for: Statistical shape modeling of the proximal femur in Mexican women: a cross-sectional morphometric and densitometric study
Source: Arch Osteoporos. 2026 Apr 24;21(1):71. doi: 10.1007/s11657-026-01682-0 (PMC13109141; doi:10.1007/s11657-026-01682-0)
Supplement: Supplementary file 1 — Supplementary Material 1 (DOCX 16.0 KB) [file 11657_2026_1682_MOESM1_ESM.docx]

**Supplementary Table 1. Mean BMD values corresponding to the representative morphological patterns generated by the statistical shape model (Modes 0–5).**

For illustrative purposes, only the first five shape modes are presented, as they collectively account for approximately 80% of the total shape variability. These modes represent mean shape configurations derived from the statistical shape model and do not correspond to groups of individual participants.

|  | **General** | **Mode 0** | **Mode 1** | **Mode 2** | **Mode 3** | **Mode 4** | **Mode 5** |
| --- | --- | --- | --- | --- | --- | --- | --- |
| **BMD (g/cm²)** | | | | | | | |
| Neck | 0.971 (0.970, 0.991) | 0.970 (0.93, 1.00) | 0.965 (0.926, 1.004) | 0.904 (0.852, 0.956) | 0.973 (0.868, 1.078) | 0.925 (0.893, 1.107) | 0.903 (0.893, 1.107) |
| Upper Neck | 0.802 (0.778, 0.823) | 0.801 (0.767, 0.835) | 0.801 (0.757, 0.845) | 0.735 (0.683, 0.786) | 0.791 (0.686, 0.895) | 0.799 (0.700, 0.789) | 0.790 (0.769, 1.020) |
| Lower Neck | 1.139 (1.13, 1.15) | 1.139 (1.103, 1.175) | 1.128 (1.092, 1.164) | 1.073 (1.015, 1.131) | 1.112 (1.04, 1.25) | 1.174 (1.076, 1.271) | 1.192 (1.06, 1.321) |
| Ward’s Triangle | 0.785 (0.761, 0.809) | 0.791 (0.754, 0.829) | 0.779 (0.732, 0.827) | 0.721 (0.666, 0.776) | 0.766 (0.675, 0.889) | 0.768 (0.667, 0.869) | 0.787 (0.749, 0.995) |
| Trochanter | 0.772 (0.760, 0.799) | 0.783 (0.755, 0.812) | 0.774 (0.748, 0.822) | 0.718 (0.656, 0.780) | 0.736 (0.661, 0.835) | 0.742 (0.712, 0.772) | 0.728 (0.649, 0.995) |
| Diaphysis | 1.228 (1.209, 1.259) | 1.238 (1.200, 1.276) | 1.227 (1.183, 1.278) | 1.174 (1.095, 1.254) | 1.191 (1.082, 1.324) | 1.195 (1.124, 1.266) | 1.190 (1.166, 1.472) |
| Total | 1.015 (0.99, 1.03) | 1.017 (0.985, 1.049) | 1.010 (0.975, 1.057) | 0.951 (0.887, 1.014) | 0.981 (0.891, 1.098) | 1.005 (0.934, 1.137) | 1.012 (0.976, 1.229) |
