# Supplementary material for: Statistical shape modeling of the proximal femur in Mexican women: a cross-sectional morphometric and densitometric study
Source: Arch Osteoporos. 2026 Apr 24;21(1):71. doi: 10.1007/s11657-026-01682-0 (PMC13109141; doi:10.1007/s11657-026-01682-0)
Supplement: Supplementary file 2 — Supplementary Material 2 (DOCX 25.2 KB) [file 11657_2026_1682_MOESM2_ESM.docx]

**Supplementary table 2. Complete linear regression results between shape modes (HSM 0–16) and regional BMD.**

The table presents regression coefficients (β), standard errors (SE), 95% confidence intervals, p-values, and R² for each combination of shape mode and BMD region (total, femoral neck, upper neck, lower neck, Ward’s triangle, trochanter, and shaft). Analyses were performed for exploratory purposes to describe preliminary morphometric trends in this cross-sectional study.

| **BMD Region** | **Mode** | **β** | **SE** | **95% CI** | **p-value** | **R²** |
| --- | --- | --- | --- | --- | --- | --- |
| **Total BMD** | Mode 0 | -0.59 | 0.24 | –1.07 to –0.11 | 0.012 | 0.02 |
|  | Mode 1 | -0.79 | 0.28 | –1.34 to –0.24 | 0.002 | 0.16 |
|  | Mode 2 | -0.06 | 0.40 | –0.84 to +0.72 | 0.083 | 0.01 |
|  | Mode 3 | -0.76 | 0.47 | –1.66 to +0.14 | 0.081 | 0.09 |
|  | Mode 4 | 0.24 | 0.50 | –0.74 to +1.22 | 0.079 | 0.02 |
|  | Mode 5 | 0.40 | 0.62 | –0.82 to +1.62 | 0.087 | 0.03 |
|  | Mode 6 | 1.27 | 0.78 | –0.27 to +2.81 | 0.086 | 0.09 |
|  | Mode 7 | 0.89 | 0.86 | –0.79 to +2.57 | 0.083 | 0.04 |
|  | Mode 8 | 2.36 | 0.87 | +0.65 to +4.07 | 0.004 | 0.02 |
|  | Mode 9 | -1.76 | 0.97 | –3.66 to +0.14 | 0.084 | 0.01 |
|  | Mode 10 | -2.23 | 1.06 | –4.31 to –0.15 | 0.032 | 0.01 |
|  | Mode 11 | -0.55 | 1.13 | –2.76 to +1.66 | 0.099 | 0.01 |
|  | Mode 12 | 2.28 | 1.20 | –0.07 to +4.63 | 0.050 | 0.01 |
|  | Mode 13 | 0.30 | 1.25 | –2.15 to +2.75 | 0.074 | 0.01 |
|  | Mode 14 | 2.79 | 1.37 | +0.11 to +5.47 | 0.042 | 0.01 |
|  | Mode 15 | 1.9 | 1.47 | –0.98 to +4.78 | 0.072 | 0.01 |
|  | Mode 16 | 0.24 | 0.50 | –0.74 to +1.22 | 0.071 | 0.02 |
| **Neck BMD** | Mode 0 | -0.65 | 0.28 | -1.20 to -0.10 | 0.025 | 0.02 |
|  | Mode 1 | -0.78 | 0.31 | -1.39 to -0.17 | 0.015 | 0.02 |
|  | Mode 2 | -0.19 | 0.47 | -1.11 to 0.73 | 0.083 | 0.01 |
|  | Mode 3 | -1.21 | 0.54 | -2.27 to -0.15 | 0.024 | 0.02 |
|  | Mode 4 | 1.10 | 0.56 | 0.00 to 2.20 | 0.058 | 0.01 |
|  | Mode 5 | 0.36 | 0.68 | -0.97 to 1.69 | 0.076 | 0.01 |
|  | Mode 6 | 0.97 | 0.88 | -0.75 to 2.69 | 0.089 | 0.01 |
|  | Mode 7 | -0.01 | 0.96 | -1.89 to 1.87 | 0.087 | 0.01 |
|  | Mode 8 | 0.97 | 1.02 | -1.03 to 2.97 | 0.082 | 0.01 |
|  | Mode 9 | -2.04 | 1.11 | -4.22 to 0.14 | 0.081 | 0.01 |
|  | Mode 10 | -2.25 | 1.20 | -4.60 to 0.10 | 0.084 | 0.01 |
|  | Mode 11 | -1.31 | 1.37 | -4.00 to 1.38 | 0.086 | 0.02 |
|  | Mode 12 | 3.44 | 1.45 | 0.60 to 6.28 | 0.011 | 0.02 |
|  | Mode 13 | -0.88 | 1.43 | -3.68 to 1.92 | 0.101 | 0.01 |
|  | Mode 14 | 0.83 | 1.53 | -2.17 to 3.83 | 0.083 | 0.01 |
|  | Mode 15 | 3.08 | 1.64 | -0.13 to 6.29 | 0.092 | 0.01 |
|  | Mode 16 | -0.20 | 1.62 | -3.38 to 2.98 | 0.093 | 0.01 |
| **Upper Neck** | Mode 0 | -0.66 | 0.30 | -1.25 to -0.07 | 0.214 | 0.02 |
|  | Mode 1 | -0.84 | 0.33 | -1.49 to -0.19 | 0.018 | 0.03 |
|  | Mode 2 | -0.26 | 0.50 | -1.24 to 0.72 | 0.096 | 0.01 |
|  | Mode 3 | -1.34 | 0.57 | -2.46 to -0.22 | 0.024 | 0.02 |
|  | Mode 4 | 1.74 | 0.59 | 0.58 to 2.90 | 0.003 | 0.03 |
|  | Mode 5 | 0.23 | 0.73 | -1.20 to 1.66 | 0.082 | 0.01 |
|  | Mode 6 | 0.64 | 0.94 | -1.20 to 2.48 | 0.101 | 0.01 |
|  | Mode 7 | -0.11 | 1.02 | -2.11 to 1.89 | 0.083 | 0.01 |
|  | Mode 8 | 1.27 | 1.09 | -0.87 to 3.41 | 0.084 | 0.01 |
|  | Mode 9 | -2.87 | 1.17 | -5.16 to -0.58 | 0.019 | 0.02 |
|  | Mode 10 | -2.10 | 1.28 | -4.61 to 0.41 | 0.088 | 0.01 |
|  | Mode 11 | -0.64 | 1.46 | -3.50 to 2.22 | 0.087 | 0.01 |
|  | Mode 12 | 4.55 | 1.53 | 1.55 to 7.55 | 0.098 | 0.04 |
|  | Mode 13 | -1.43 | 1.52 | -4.41 to 1.55 | 0.054 | 0.01 |
|  | Mode 14 | 0.32 | 1.83 | -3.27 to 3.91 | 0.052 | 0.01 |
|  | Mode 15 | 3.86 | 1.74 | 0.45 to 7.27 | 0.026 | 0.02 |
|  | Mode 16 | -0.74 | 1.72 | -4.11 to 2.63 | 0.058 | 0.01 |
| **Lower Neck** | Mode 0 | -0.63 | 0.28 | -1.18 to -0.08 | 0.026 | 0.02 |
|  | Mode 1 | -0.71 | 0.31 | -1.32 to -0.10 | 0.025 | 0.02 |
|  | Mode 2 | -0.11 | 0.47 | -1.03 to 0.81 | 0.058 | 0.01 |
|  | Mode 3 | -1.03 | 0.54 | -2.09 to 0.03 | 0.094 | 0.01 |
|  | Mode 4 | 0.47 | 0.57 | -0.65 to 1.59 | 0.088 | 0.01 |
|  | Mode 5 | 0.54 | 0.69 | -0.81 to 1.89 | 0.109 | 0.01 |
|  | Mode 6 | 0.29 | 0.89 | -1.45 to 2.03 | 0.101 | 0.01 |
|  | Mode 7 | 0.09 | 0.97 | -1.81 to 1.99 | 0.104 | 0.01 |
|  | Mode 8 | 0.72 | 1.03 | -1.30 to 2.74 | 0.095 | 0.01 |
|  | Mode 9 | -1.17 | 1.12 | -3.37 to 1.03 | 0.086 | 0.01 |
|  | Mode 10 | -2.43 | 1.21 | -4.80 to -0.06 | 0.044 | 0.01 |
|  | Mode 11 | -1.92 | 1.37 | -4.61 to 0.77 | 0.097 | 0.01 |
|  | Mode 12 | 2.19 | 1.46 | -0.67 to 5.05 | 0.088 | 0.01 |
|  | Mode 13 | -0.35 | 1.44 | -3.17 to 2.47 | 0.085 | 0.01 |
|  | Mode 14 | 1.37 | 1.54 | -1.65 to 4.39 | 0.086 | 0.01 |
|  | Mode 15 | 2.43 | 1.65 | -0.80 to 5.66 | 0.083 | 0.01 |
|  | Mode 16 | 0.29 | 1.62 | -2.89 to 3.47 | 0.084 | 0.01 |
| **Ward’s Triangle** | Mode 0 | -1.16 | 0.27 | -1.69 to -0.63 | 0.006 | 0.06 |
|  | Mode 1 | -0.66 | 0.32 | -1.29 to -0.03 | 0.042 | 0.01 |
|  | Mode 2 | 0.11 | 0.46 | -0.79 to 1.01 | 0.081 | 0.01 |
|  | Mode 3 | -1.48 | 0.53 | -2.52 to -0.44 | 0.004 | 0.02 |
|  | Mode 4 | -0.21 | 0.57 | -1.33 to 0.91 | 0.083 | 0.01 |
|  | Mode 5 | 0.09 | 0.71 | -1.30 to 1.48 | 0.084 | 0.01 |
|  | Mode 6 | 2.22 | 0.88 | 0.50 to 3.94 | 0.015 | 0.02 |
|  | Mode 7 | -0.16 | 0.99 | -2.10 to 1.78 | 0.085 | 0.01 |
|  | Mode 8 | 1.91 | 1.00 | -0.05 to 3.87 | 0.056 | 0.01 |
|  | Mode 9 | -3.47 | 1.09 | -5.61 to -1.33 | 0.004 | 0.03 |
|  | Mode 10 | -1.76 | 1.22 | -4.15 to 0.63 | 0.054 | 0.01 |
|  | Mode 11 | -0.66 | 1.29 | -3.19 to 1.87 | 0.086 | 0.01 |
|  | Mode 12 | 2.81 | 1.37 | 0.12 to 5.50 | 0.044 | 0.01 |
|  | Mode 13 | -0.02 | 1.43 | -2.82 to 2.78 | 0.087 | 0.01 |
|  | Mode 14 | 0.68 | 1.57 | -2.40 to 3.76 | 0.088 | 0.01 |
|  | Mode 15 | 3.34 | 1.67 | 0.07 to 6.61 | 0.049 | 0.01 |
|  | Mode 16 | -0.57 | 1.70 | -3.90 to 2.76 | 0.098 | 0.01 |
| **Trochanter** | Mode 0 | -0.43 | 0.22 | -0.86 to 0.00 | 0.042 | 0.01 |
|  | Mode 1 | -0.47 | 0.25 | -0.96 to 0.02 | 0.081 | 0.01 |
|  | Mode 2 | -0.48 | 0.36 | -1.19 to 0.23 | 0.081 | 0.01 |
|  | Mode 3 | -0.13 | 0.42 | -0.95 to 0.69 | 0.085 | 0.01 |
|  | Mode 4 | 0.31 | 0.45 | -0.57 to 1.19 | 0.083 | 0.01 |
|  | Mode 5 | 0.68 | 0.56 | -0.42 to 1.78 | 0.084 | 0.01 |
|  | Mode 6 | 0.90 | 0.70 | -0.47 to 2.27 | 0.096 | 0.01 |
|  | Mode 7 | 0.95 | 0.77 | -0.56 to 2.46 | 0.109 | 0.01 |
|  | Mode 8 | 2.44 | 0.78 | 0.91 to 3.97 | 0.001 | 0.03 |
|  | Mode 9 | -1.27 | 0.87 | -2.98 to 0.44 | 0.094 | 0.01 |
|  | Mode 10 | -0.92 | 0.96 | -2.80 to 0.96 | 0.095 | 0.01 |
|  | Mode 11 | 0.22 | 1.01 | -1.76 to 2.20 | 0.093 | 0.01 |
|  | Mode 12 | 2.13 | 1.07 | 0.03 to 4.23 | 0.086 | 0.01 |
|  | Mode 13 | 0.51 | 1.12 | -1.69 to 2.71 | 0.089 | 0.01 |
|  | Mode 14 | 2.56 | 1.22 | 0.17 to 4.95 | 0.088 | 0.01 |
|  | Mode 15 | 1.31 | 1.31 | -1.26 to 3.88 | 0.089 | 0.01 |
|  | Mode 16 | 2.14 | 1.33 | -0.47 to 4.75 | 0.087 | 0.01 |
| **Diaphysis** | Mode 0 | -0.17 | 0.28 | -0.72 to 0.38 | 0.101 | 0.01 |
|  | Mode 1 | -1.16 | 0.32 | -1.79 to -0.53 | 0.006 | 0.04 |
|  | Mode 2 | 0.35 | 0.47 | -0.57 to 1.27 | 0.102 | 0.01 |
|  | Mode 3 | -1.26 | 0.55 | -2.34 to -0.18 | 0.024 | 0.01 |
|  | Mode 4 | 0.45 | 0.59 | -0.71 to 1.61 | 0.096 | 0.01 |
|  | Mode 5 | 0.34 | 0.73 | -1.09 to 1.77 | 0.073 | 0.01 |
|  | Mode 6 | 1.71 | 0.91 | -0.07 to 3.49 | 0.095 | 0.01 |
|  | Mode 7 | 1.21 | 1.01 | -0.77 to 3.19 | 0.096 | 0.01 |
|  | Mode 8 | 3.15 | 1.02 | 1.15 to 5.15 | 0.004 | 0.03 |
|  | Mode 9 | -1.84 | 1.13 | -4.05 to 0.37 | 0.094 | 0.01 |
|  | Mode 10 | -3.34 | 1.24 | -5.77 to -0.91 | 0.004 | 0.02 |
|  | Mode 11 | -0.84 | 1.32 | -3.43 to 1.75 | 0.104 | 0.01 |
|  | Mode 12 | 1.91 | 1.41 | -0.85 to 4.67 | 0.096 | 0.01 |
|  | Mode 13 | 0.55 | 1.46 | -2.31 to 3.41 | 0.093 | 0.01 |
|  | Mode 14 | 4.00 | 1.59 | 0.88 to 7.12 | 0.012 | 0.02 |
|  | Mode 15 | 2.50 | 1.71 | -0.85 to 5.85 | 0.094 | 0.01 |
|  | Mode 16 | 2.42 | 1.73 | -0.97 to 5.81 | 0.095 | 0.01 |
